# Decomposing the Poor-Non-Poor Gap in the Prevalence of Undiagnosed and Untreated Hypertension Among Bangladeshi Population

**DOI:** 10.5334/gh.1372

**Published:** 2024-12-05

**Authors:** Mosiur Rahman, Mahfuza Khatun, Asrafun Naher Pinkey, Syed Emdadul Haque, Farhana Akhter Liza, Md. Nuruzzaman Haque, Prosannajid Sarkar, Tapan Kumar Roy, G. M. Rabiul Islam, Md. Rashed Alam, Mahmudul Hasan, Izzeldin Fadl Adam, Nguyen Huu Chau Duc, Saber Al-Sobaihi, Abid Hasan

**Affiliations:** 1 Department of Population Science and Human Resource Development, University of Rajshahi, Rajshahi-6205, Bangladesh; 2 Uchicago Research Bangladesh, Dhaka, Bangladesh; 3 Dr. Wazed Research and Training Institute, Begum Rokeya University, Rangpur, Bangladesh; 4 Department of Food Engineering and Tea Technology, Shahjalal University of Science and Technology, Sylhet, 3114, Bangladesh; 5 Faculty of Public Health, University of Khartoum, Sudan; 6 Hue University of Medicine and Pharmacy, Hue University, Vietnam; 7 Premium Research Institute for Human Metaverse Medicine (WPI-PRIMe), Osaka University, Osaka, Japan

**Keywords:** decomposition, hypertension, undiagnosed, untreated, SES, Bangladesh

## Abstract

**Objectives:**

Our objectives were to ascertain: the prevalence and socio-economic distribution of hypertension, as well as the rates of undiagnosed and untreated hypertension; the association between socioeconomic status (SES) and the occurrence of hypertension, as well as the rates of undiagnosed and untreated hypertension; and the factors influencing the poor-non-poor gap in terms of the prevalence, diagnosis, and treatment of hypertension.

**Design:**

Cross-sectional nationally representative study.

**Methods:**

Data from the 2017–18 Bangladesh Demographic Health Survey were used. 11,776 participants who were 18 years of age or older responded to our analysis. We used the wealth index as a proxy for SES. The prevalence of hypertension, both diagnosed and undiagnosed, as well as its untreated states, were the outcome variables.

**Results:**

The age-adjusted prevalence of hypertension, undiagnosed as having hypertension, and untreated cases were 25.1%, 57.2%, and 12.3%, respectively. People in the poor SES groups had a 0.88 times (95% confidence interval [CI] 0.77–0.99) lower likelihood of having hypertension compared to those in the non-poor SES group. Individuals belonging to the poor SES group exhibited a likelihood of 1.68 and 1.53 times greater for having untreated hypertension and being undiagnosed with the condition, respectively, compared to those in the non-poor SES group. The results indicated that BMI played a role in increasing the disparity between the poor and non-poor populations concerning hypertension risk. Additionally, factors such as age, gender, and education were found to exacerbate the gap in the risk of undiagnosed hypertension between these two groups.

**Conclusion:**

The results of this study suggest that appropriate policy measures be developed for ongoing care and early identification, especially for older adults, men, and individuals with low levels of education from low socioeconomic backgrounds. Additionally, efforts must be made to reduce the prevalence of overweight and obesity among people in the non-poor SES category.

## 1. Introduction

One of the most preventable causes of early death and disability is hypertension, a major non-communicable disease (NCD) (1). Over 10.4 million deaths and 218 million disability-adjusted life years were caused by hypertension worldwide in 2017 (2). Undiagnosed hypertension poses a further risk to health because many people do not take medication to treat their hypertension. Globally, an estimated 46% of adults with hypertension are unaware that they have the condition, and less than half of adults (42%) with hypertension are diagnosed and treated (3). To create health promotion and disease prevention programs, there is a pressing need for improved hypertension screening and early identification, given the significant global burden of undiagnosed and untreated hypertension (4).

The World Health Organization’s worldwide report on hypertension stated that the prevalence of hypertension among adults in South Asia aged 30–79 is 36.5%, which is marginally higher than the global prevalence of 33% in the year 2019 (5). Bangladesh, a country in South Asia, has the lowest prevalence when compared to other countries in the region, yet a higher percentage of people between the ages of 30–79 (29%) have hypertension (5). In Bangladesh, the weighted pooled prevalence of hypertension is 20%, ranging from 1.1% to 75%, per a systematic review investigation (6). This discrepancy in values between the pooled prevalence estimates could be attributed to publication bias, evidence of small study effects, heterogeneity in the universal definition of hypertension, and differences in the age of study participants.

Data on undiagnosed and untreated hypertension and its associated causes are not well organized or synthesized, especially in Bangladesh. The prevalence of undiagnosed hypertension among adults 35 years of age and older was reported to be 45% and 82%, respectively, in two small-scale local investigations conducted in rural Bangladesh (7,8). 48.9% and 49.9% of Bangladeshis, respectively, had untreated hypertension and were undiagnosed, according to a nationwide 2011 study (9). However, as the participants in these studies were restricted to individuals 35 years of age or older, the data cannot adequately represent the current condition of hypertension since the disease has been concentrating in younger individuals in recent years (10).

Among several sociodemographic, behavioral, and health-related factors that affect the prevalence, diagnosis, and treatment of hypertension (7,8,9,10,11,12,13), socioeconomic status (SES) is one of the most important independent variables. Surprisingly, SES discrepancies have not received much attention in attempts to prevent and control hypertension in low- and middle-income countries (LMICs), despite research on SES inequality relating to hypertension prevalence, diagnosis, and treatment having been done in several high-income nations (14,15,16,17). Studies show that hypertension is more prevalent in poor SES groups in high-income countries (14,15,16,17). On the other hand, in contrast to high-income nations, hypertension is more prevalent in the high SES group in LMICs (18,19).

Bangladesh’s national health policy to prevent and provide access to care for hypertension does not include an action plan to address socioeconomic inequities. It is critical to gain a deeper understanding of the socially disadvantaged populations that face the biggest obstacles to receiving a diagnosis and treatment for hypertension in low-resource environments like Bangladesh, where rapid industrialization and urbanization over the past few decades have increased SES inequalities (20). However, no study has yet used the most recent data from the Bangladesh Demographic and Health Survey (BDHS), which includes the years 2017–2018, to investigate socioeconomic variations in undiagnosed and untreated hypertension. Furthermore, no research has yet been done on the causes of the disparity between the poor and non-poor in the prevalence, diagnosis, and treatment of hypertension in Bangladesh. The objectives of this study are to investigate: the prevalence and socio-economic distribution of hypertension, undiagnosed as having hypertension, and untreated for hypertension; the relationship between SES and the prevalence of hypertension, undiagnosed as having hypertension, and untreated for hypertension; and the factors influencing the disparity between the poor and non-poor in terms of the prevalence, diagnosis, and treatment of hypertension.

## 2. Methods

### 2.1 Data source

The most recent wave of data from the Bangladesh Demographic and Health Survey (BDHS) 2017–18 was examined in the current study (21). Data were accessed for research purposes on 01/08/2023. The BDHS gathers data on matters pertaining to population health and demography through periodic population-based surveys that are nationally representative. The survey was conducted between October 2017 and March 2018 under the auspices of the Ministry of Health and Family Welfare, Medical Education and Family Welfare Division, and National Institute of Population Research and Training. The main goals of the survey were to evaluate the health indicators, give a thorough overview of demographic, maternal, and child health issues, and measure the prevalence of numerous NCDs like adult hypertension (21).

### 2.2 Study population and survey design

The BDHS employed a stratified two-stage sample of dwellings, with distinct strata for rural and urban areas. Primarily, primary sampling units (PSUs) were identified using enumeration areas from the 2011 Bangladesh census; PSUs are groups of houses with an average size of 120. In the first stage, 675 PSUs were randomly selected from a total of 293,579 PSUs. PSUs totaled 672 (192 in urban and 480 in rural areas). The remaining three PSUs weren’t sampled because of the flooding. For the second step, a sample of 20,160 households was selected, 30 from each selected PSU, to collect data. 96.5% of them got their interviews completed, or 19,457 households.

**Figure 1:**
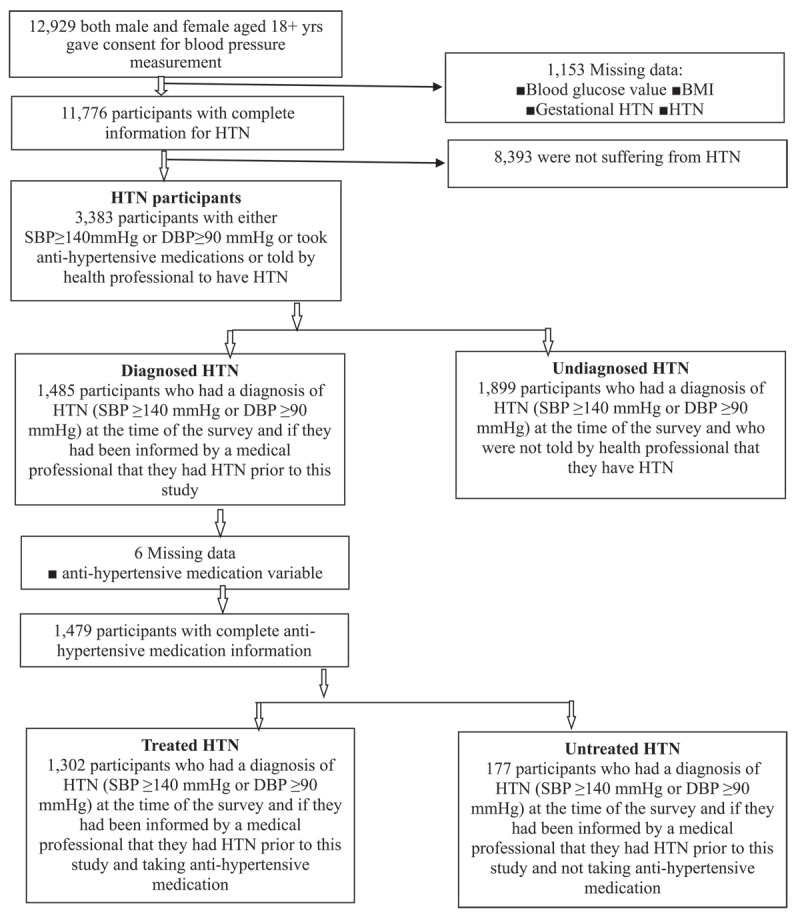
Selection of the sample.

The BDHS 2017–18 collected blood pressure (BP) readings from 4,864 households, or one-fourth of the selected households (7–8 homes in each cluster). 12,299 men and women who were at least 18 years old participated in BP measurement interviews among those selected households. Figure 1 shows a detailed flowchart for the study participant selection process.

### 2.3 Measures

#### 2.3.1 Outcome measures

The prevalence of hypertension, both diagnosed and undiagnosed, as well as its untreated states, piqued our attention. The 2017–18 BDHS used the LIFE SOURCE^®^ UA-767 Plus Blood Pressure Monitor model in accordance with the World Health Organization’s (WHO) recommended approach for measuring hypertension (21). Participants in the survey who were seated had their blood pressure taken in their right arm. Throughout the survey, three readings of the patient’s systolic and diastolic blood pressure were obtained, spaced about ten minutes apart. The respondent’s blood pressure levels were reported using the average of the second and third measurements. According to the survey, systolic and diastolic blood pressure were recorded in millimeters of mercury [mmHg].

A person was classified as hypertensive if their diastolic blood pressure (DBP) was ≥90 mmHg, their systolic blood pressure (SBP) was ≥140 mmHg, and/or they were using any anti-hypertensive drugs to lower their blood pressure or were previously diagnosed as hypertensive by any health professional. We classified hypertension using the BP cut-off value of 140/90 mmHg in accordance with the National Guidelines for Management of Hypertension in Bangladesh (22), which are in line with the 2018 European Society of Hypertension and European Society of Cardiology hypertension recommendations (23).

A participant was undiagnosed as having hypertension if, at the time of the survey, their blood pressure was diagnosed at ≥140 mmHg or DBP ≥90 mmHg, but they had never taken any prescribed anti-hypertensive medication to lower or control their blood pressure, or if they had never received a diagnosis of hypertension from a medical professional before this study. A participant was deemed to be untreated for hypertension if, at the time of the survey, their blood pressure was diagnosed as hypertensive (SBP ≥ 140 mmHg or DBP ≥ 90 mmHg), they had been informed by a healthcare provider that they had high blood pressure prior to this study, and they had not taken any prescribed anti-hypertensive medication to lower or control their blood pressure.

#### 2.3.2 Exposure

Using a wealth index as a proxy for SES, we calculated the gap in the prevalence of hypertension, the number of cases of undiagnosed hypertension, and the number of cases of untreated hypertension between the poor and the non-poor. Based on data regarding household assets, such as having durable items like TVs and bicycles and dwellings (including a supply of drinking water, sanitizing facilities, and construction supplies), the BDHS wealth index was calculated (21). A weight, or factor score, was given to every asset based on principal component analysis. Subsequently, the asset ratings were normalized using the standard normal distribution, which has a standard deviation of one and a mean of zero. Each household was assigned a score for each asset, and the scores were then totaled. Subsequently, the sample was divided into population quintiles and assigned a grade of zero for the poorest, one for the poorer, three for the middle, four for the richer, or five for the richest. People were ranked based on the total score of the household in which they lived. We labeled the top three quintiles of the wealth index as ‘non-poor’ and the bottom two as ‘poor’ in our analysis. This classification coincides with previous studies (24,25,26).

#### 2.3.3 Covariates

Several socioeconomic and demographic characteristics that have been theoretically and empirically linked to the prevalence, diagnosis, and treatment of hypertension were considered in this study (7,8,9,10,11,12,13,14,15,16,17,18,19). 18–34, 35–39, 40–44, 45–49, 50–54, 55–59, 60–64, and 65 and older were the age groups into which the responders were divided. Due to the long-held belief that hypertension is an adult-onset disease (27) and the evidence supporting the significant increase in the risk of developing the condition beyond that age, we first divided the population into those between the ages of 18–34. At a five-year interval, other groups were then taken into consideration because the trends were frequently consistent across all age groups. Based on Bangladesh’s official educational system, the respondents’ educational attainment levels were divided into four groups: no education (0 years), primary education (1–5 years), secondary education (6–10 years), and higher education (11 years and above). Families headed by patriarchs and non-patriarchs were distinguished among the respondents.

The type of residence was identified as either rural or urban. The respondents’ current marital status and employment status were identically divided into yes/no categories. Tercile was utilized to identify the number of adults in the family and family sizes in our study sample. The weight in kilograms multiplied by the squared height in meters (kg/m^2^) yielded the body mass index (BMI). A BMI of less than 18.5 kg/m^2^ was considered underweight, 18.5–24.99 kg/m^2^ was considered normal, 25–29.99 kg/m^2^ was considered overweight, and 30 kg/m^2^ or over was classified as obesity, according to the 2017–2018 BDHS report (21).

### 2.4 Statistical analyses

We calculated the age-adjusted prevalence of hypertension (diagnosed + undiagnosed) in individuals using a direct standardization approach. For the age-adjusted prevalence estimates, we created a reference population using the age-specific population of Bangladeshis 18 and older from the 2011 census. To calculate the age-adjusted prevalence of undiagnosed and untreated hypertension, we used the age distribution of hypertension in the research participants as a reference group. Bivariate distributions were displayed to observe the sociodemographic differences in the prevalence of hypertension (diagnosed + undiagnosed), undiagnosed as having hypertension, and untreated for hypertension. Logistic regression models were employed to investigate the association between SES and the prevalence of hypertension, undiagnosed as having hypertension and untreated for hypertension, after accounting for other independent variables. All the covariates were included simultaneously in the multiple regression models. Finding that the variance inflation factors were 2.0, it was feasible to draw the conclusion that multicollinearity was absent. To evaluate the strength of the relationships, we calculated the odds ratios and performed a significant test using the 95% confidence intervals (CIs). A significant threshold of *P* < 0.05 was chosen.

To investigate the degree to which compositional variations in sociodemographic factors contributed to the poor-non-poor gap between hypertension, undiagnosed as having hypertension, and untreated for hypertension, the non-linear Fairlie decomposition (28) approach was utilized. Since the dependent variables were binary, we used Fairlie’s nonlinear decomposition techniques. The results of the study were presented using guidelines to strengthen the reporting of observational studies in epidemiology. Stata version 15.0 (StataCorp. LP, College Station, USA) was used for all analyses to account for sample weights based on the complex survey design of the BDHS.

### 2.5 Ethical considerations

The ORC macro institutional review board gave their approval to the methods utilized in the BDHS data collection. The protocol for the survey was reviewed and approved by the Ministry of Health and Family Welfare of Bangladesh’s National Ethics Review Committee. The informed consent was given by each respondent before commencing the interview. Subsequently, the interviewers gave an oral explanation compliant with BDHS principles. Since the study’s basis was anonymous secondary data without any personally identifiable information on the survey respondents, it was excused from an exhaustive assessment. Every study protocol was followed in accordance with the 2013 revision of the Declaration of Helsinki’s guiding principles.

## 3. Results

### 3.1 Descriptive statistics

Table 1 displays the sociodemographic attributes of the participants. 11,776 people in all participated in this study. 56.2% of the respondents were female, 80.5% were married, and 44% of the respondents were between the ages of 18 and 34. Approximately 62.1% of respondents said they were employed, 73.5% of respondents stated they were living in rural areas, 80.5% reported they were married, and about 26% reported they had no formal education.

**Table 1:** Descriptive statistics according to the age-adjusted prevalence of hypertension, undiagnosed hypertension, and untreated hypertension among individuals aged 18 years or older: 2017–2018, Bangladesh Demographic and Health Survey.


MEASURES	%^1^	AGE-ADJUSTED PREVALENCE (%)		

		HYPERTENSION (n = 11,776)	UNDIAGNOSED HYPERTENSION (n = 3,383)	UNTREATED HYPERTENSION (n = 1,479)

Age, yrs				

18–34	43.8	13	70.9	24.4

35–39	11.8	27.6	68	12.3

40–44	8.9	30.7	55.8	15.6

45–49	8.5	37.2	53.9	7.2

50–54	5.7	41.2	53.5	13.1

55–59	5.8	45.1	46.7	7.5

60–64	5.8	49.8	45.7	7.3

65+	9.6	55.4	51.8	11

Currently married				

No	19.5	25	62.1	11.5

Yes	80.5	24.7	56.9	12.5

Currently working				

No	37.9	28.6	50.2	10.4

Yes	62.1	23.4	62.5	14.2

BMI				

Underweight	17.6	13.3	70.6	15.2

Normal	58.4	22.2	60.6	11.7

Overweight/obese	24.1	40.4	47.5	12.4

Education				

No education	26	23.3	60.8	65.4

Primary	30.1	24.3	54.8	61.6

Secondary	29	27.8	55.6	60

Higher	14.9	29.6	55.6	61.3

Gender				

Female	56.2	27.8	50.1	11.4

Male	43.8	22.3	67.4	14.1

Living in a patriarchal family				

No	11.9	27.7	54.4	10.6

Yes	88.1	24.8	57.6	12.5

No. of adult member				

1–2	36.3	25.6	60	14.2

3	25	25.6	58.3	9.4

4+	38.7	24.6	54.5	12.7

No. of household member				

1–4	43.6	25.8	59.1	13.4

5	19.3	25.7	55.6	8.7

6+	37.1	24.1	55.6	12.9

Place of residence				

Rural	73.5	24.3	59.1	13.4

Urban	26.5	27.6	51.7	9.7

Division				

Barisal	5.6	27.6	56.7	12.1

Chittagong	17.2	28.5	52.6	7.9

Dhaka	22.3	22.6	54.2	7.7

Khulna	12.6	25.7	56.5	15.6

Mymensingh	8.2	20	58.1	8.1

Rajshahi	14.7	25.1	59.9	21.3

Rangpur	12.8	27.4	66.9	20.9

Sylhet	6.6	23.9	49.7	5.3

SES				

Nonpoor	60.6.	27.4	52.6	11

Poor	39.4	21.7	66.3	15.5

**Prevalence**		25.1	57.2	12.3


Note:^1^ Weighted percentage was presented.

According to BMI estimations, 58.4% of people were of normal weight, 17.6% were underweight, and 24.1% were overweight or obese. Furthermore, 39.4% of respondents belonged to the poor SES group, and 88.1% of respondents lived in patriarchal families. The age-adjusted prevalence of hypertension in our sample was 25.1%. Age-adjusted prevalence of undiagnosed cases of hypertension was 57.2% among the hypertensive patients. In our sample data, the age-adjusted prevalence of untreated cases among the hypertension patients with a diagnosis was 12.3%.

Table 1 also displays the sociodemographic variations in the prevalence of hypertension, undiagnosed as having hypertension, and untreated for hypertension. Compared to the poor SES group (21.7%), the non-poor SES groups had a greater prevalence of hypertension (27.4%). On the other hand, we observed the reverse situation when it came to undiagnosed as having hypertension: the poor group (66.3%) had greater rates of undiagnosed as having hypertension than the non-poor SES group (52.6%). Untreated hypertension showed similar trends, with the poor SES groups having higher rates of untreated hypertension (15.5%) compared to the non-poor SES group (11%).

### 3.2 Multivariable analyses

#### 3.2.1 Association between hypertension, undiagnosed hypertension, and untreated hypertension with SES and other covariates

Table 2 displays the aORs of the association between SES and the prevalence of hypertension, undiagnosed as having hypertension, and untreated for hypertension in our study group. People in the poor SES group had a 0.88 times (95% CI 0.77–0.99) lower likelihood of getting hypertension than those in the non-poor SES group. The odds of undiagnosed as having hypertension were 1.57 times (95% CI 1.28–1.93) higher for those in the poor SES groups than for those in the non-poor SES group. The likelihood of untreated hypertension was 1.68 times (95% CI 1.02–2.76) higher in the poor SES groups compared to the non-poor SES group.

**Table 2:** Adjusted odds ratio for the association between SES and other covariates with the prevalence of hypertension, undiagnosed hypertension, and untreated hypertension among individuals aged 18 years or older: 2017–2018, Bangladesh Demographic and Health Survey.


MEASURES	aOR (95% CI)		

	HYPERTENSION (n = 11,776)	UNDIAGNOSED HYPERTENSION (n = 3,383)	UNTREATED HYPERTENSION (n = 1,479)

Age, yrs			

18–34	1.00	1.00	1.00

35–39	2.54 (2.13–3.02)*^a^*	0.90 (0.64–1.26)	0.44 (0.23–0.88)*^c^*

40–44	3.11 (2.59–3.74)*^a^*	0.51 (0.37–0.70)*^a^*	0.60 (0.32–1.12)

45–49	4.26 (3.50–5.19)*^a^*	0.48 (0.35–0.66)*^a^*	0.25 (0.13–0.50)*^a^*

50–54	5.89 (4.75–7.29)*^a^*	0.37 (0.27–0.51)*^a^*	0.44 (0.23–0.85)*^c^*

55–59	6.99 (5.73–8.53)*^a^*	0.27 (0.20–0.39)*^a^*	0.25 (0.12–0.52)*^a^*

60–64	9.03 (7.31–11.17)*^a^*	0.23 (0.17–0.32)*^a^*	0.25 (0.12–0.49)^a^

65+	12.03 (9.94–14.56)*^a^*	0.26 (0.19–0.35)*^a^*	0.36 (0.20–0.64)*^b^*

Currently married			

No	1.00	1.00	1.00

Yes	0.78 (0.67–0.91)*^c^*	0.74 (0.59–0.92)*^b^*	0.71 (0.43–1.18)

Currently working			

No	1.00	1.00	1.00

Yes	0.92 (0.81–1.05)	1.05 (0.87–1.28)	1.21 (0.79–1.85)

BMI			

Underweight	1.00	1.00	1.00

Normal	1.93 (1.69–2.21)*^a^*	0.63 (0.49–0.83)*^b^*	0.85 (0.43–1.69)

Overweight/obese	5.04 (4.27–5.95)*^a^*	0.44 (0.33–0.59)*^a^*	0.89 (0.43–1.82)

Education			

No education	1.00	1.00	1.00

Primary	1.06 (0.93–1.21)	0.79 (0.64–0.98)*^c^*	1.57 (0.99–2.48)

Secondary	1.12 (0.96–1.31)	0.81 (0.63–1.05)	1.10 (0.63–1.91)

Higher	1.13 (0.94–1.36)	0.82 (0.60–1.29)	1.82 (0.94–3.52)

Gender			

Female	1.00	1.00	1.00

Male	0.80 (0.71–0.91)*^a^*	2.36 (1.92–2.90)*^a^*	1.19 (0.77–1.86)

Living in a patriarchal family			

No	1.00	1.00	1.00

Yes	1.05 (0.89–1.24)	0.98 (0.73–1.30)	1.35 (0.74–2.49)

No. of adult member			

1–2	1.00	1.00	1.00

3	0.95 (0.82–1.11)	1.03 (0.81–1.31)	0.89 (0.53–1.48)

4+	0.89 (0.75–1.06)	0.98 (0.76–1.27)	1.44 (0.82–2.53)

No. of household member			

1–4	1.00	1.00	1.00

5	0.99 (0.86–1.16)	0.88 (0.69–1.13)	0.57 (0.32–1.00)

6+	0.94 (0.80–1.10)	0.94 (0.74–1.21)	0.72 (0.43–1.22)

Place of residence			

Rural	1.00	1.00	1.00

Urban	1.09 (0.96–1.23)	0.89 (0.73–1.09)	0.79 (0.50–1.23)

Division			

Barisal	1.00	1.00	1.00

Chittagong	0.88 (0.70–1.11)	1.01 (0.72–1.43)	0.55 (0.25–1.20)

Dhaka	0.64 (0.51–0.81)*^a^*	1.02 (0.73–1.44)	0.61 (0.27–1.29)

Khulna	0.81 (0.65–1.02)	1.09 (0.77–1.53)	1.40 (0.65–3.01)

Mymensingh	0.67 (0.54–0.85)*^b^*	1.04 (0.72–1.50)	0.59 (0.26–1.38)

Rajshahi	0.87 (0.68–1.10)	1.10 (0.79–1.54)	1.84 (0.86–3.92)

Rangpur	1.05 (0.85–1.31)	1.40 (1.00–1.96)*^c^*	2.02 (1.03–3.97)*^c^*

Sylhet	0.86 (0.68–1.09)	0.71 (0.49–1.04)	0.37 (0.15–0.91)*^c^*

SES			

Nonpoor	1.00	1.00	1.00

Poor	0.88 (0.77–0.99)*^c^*	1.53 (1.24–1.89)*^b^*	1.68 (1.02–2.76)*^c^*


Note: CI = Confidence interval; aOR = Adjusted odds ratio. Here a, b, and c indicate *p <* 0.001, *p <* 0.01, and *p <* 0.05.

Table 2 also displays the aORs of the associations between the hypertension, undiagnosed as having hypertension, and untreated for hypertension, and other sociodemographic characteristics. Respondents who were 35–39, 40–44, 45–49, 50–54, 55–59, 60–64, and 65+ years old had considerably greater odds of developing hypertension than those who were 18–34 years old, but they also had significantly lower odds of going undiagnosed (except from the age group of 35–39 years) and untreated (except from the age group of 40–44 years). Males were less likely than females to develop hypertension, but they were also more likely to be undiagnosed as having hypertension. Normal weight and overweight/obese individuals were less likely than their counterparts to be undiagnosed as having hypertension, but they were more likely to acquire hypertension.

The likelihood of getting hypertension in the married respondents was 0.78 times (95% CI 0.67–0.91) lower than in the single respondents. The risk of being undiagnosed as having hypertension was lower in married individuals than in single individuals. The likelihood of developing hypertension was lower among respondents who resided in the divisions of Mymensingh and Dhaka. Further, respondents from the Rangpur division had 1.40- and 2.02-times increased chances of not receiving a hypertension diagnosis and treatment (Table 2).

#### 3.2.2 Decomposition of poor-non poor gaps in hypertension, undiagnosed hypertension, and untreated hypertension

Table 3 represents the effect, contribution, and the total explained gap of each predictor variable in the poor-non-poor gap in the prevalence of hypertension, the prevalence of people without a diagnosis of hypertension, and the prevalence of people with untreated hypertension. The decomposition results indicated that the observed variables were responsible for 65.1%, 30%, and 43.9% of the variations in the prevalence of hypertension, the prevalence of people without a diagnosis of hypertension, and the prevalence of people with untreated hypertension. Among the variables causing the explained part of the differences, age had a negative effect or helped to narrow the poor-non-poor gap in the prevalence of hypertension and the prevalence of people with untreated hypertension. Conversely, BMI was predicted to widen the gap in the prevalence of hypertension and contributed 133.41% to increasing the poor-non-poor gap.

**Table 3:** Decomposition results of the explained poor-nonpoor gap of hypertension, undiagnosed hypertension, and untreated hypertension among individuals aged 18 years or older: 2017–2018, Bangladesh Demographic and Health Survey.


PREDICTORS	HYPERTENSION (n = 11,776)		UNDIAGNOSED HYPERTENSION (n = 3,383)		UNTREATED HYPERTENSION (n = 1,479)	
		
	COEFFICIENT*^1^* (95%CI)	% CONTRIBUTION	COEFFICIENT*^1^* (95%CI)	% CONTRIBUTION	COEFFICIENT*^1^* (95%CI)	% CONTRIBUTION

Age	–**0.0178** **(–0.019, –0.016)**^a^	–41.01	**0.0159** **(0.013, 0.019)**^a^	43.68	**–0.0052** **(–0.008, –0.002)** ^c^	–30.2

Currently married	0.0002 (–0.0004, 0.0008)	0.44	–**0.0035** **(–0.006, –0.001)**^c^	–9.62	–0.0012 (–0.004, 0.002)	–6.97

Currently working	0.0003 (–0.0034, 0.0039)	0.64	0.0002 (–0.005, 0.006)	0.55	–0.0027 (–0.007, 0.002)	–15.78

BMI	**0.0579** **(0.0522, 0.0638)**^a^	133.41	**–0.0352** **(–0.048, –0.023)** ^a^	–96.7	–0.0048 (–0.015, 0.006)	–27.9

Education	0.0029 (–0.005, 0.11)	6.68	**0.0156** **(0.031, 0.007)**^c^	42.86	0.0023 (–0.016, 0.021)	13.37

Gender	0.0002 (–0.0008, 0.0005)	0.46	**0.0093** **(0.007, 0.012)** ^a^	21.43	0.0027 (–0.004, 0.009)	15.7

Living in a patriarchal family	–0.0008 –(0.0005, 0.0003)	–0.19	0.0009 (–0.0005, 0.0007)	0.25	0.0019 (–0.0006, 0.004)	11.04

No. of adult members	–0.0057 (–0.0105, 0.008)	–13.04	0.0013 (–0.007, 0.009)	3.57	0.0022 (–0.004, 0.008)	12.78

No. of household members	0.0005 (–0.0015, 0.0025)	1.19	–0.0016 (–0.006, 0.003)	–4.39	–0.0007 (–0.0027, 0.001)	–4.07

Place of residence	0.0051 (–0.0009, 0.011)	11.82	–0.0065 (–0.020, 0.007)	–17.86	–0.0080 (–0.019, 0.002)	–46.51

Division	–0.0009 (–0.0019, 0.0018)	–0.21	–0.0005 (–0.005, 0.004)	–1.37	–0.0033 (–0.008, 0.002)	–19.18

Total explained gap (%)*^2^*	0.0434 (65.1%)	100	–0.0364 (30%)	100	–0.0172 (43.9%)	100

Difference*^3^*	–0.0666		–0.1218		–0.0391	


Here a, b, and c indicate *p <* 0.001, *p <* 0.01, and *p <* 0.05. ^1^ A positive (negative) coefficient of the covariate indicates that it widens (reduces) the gap between the poor and the non-poor to experience the occurrence of the outcome variables. ^2^ Within the explained gap of the prevalence of hypertension, as having undiagnosed hypertension, and untreated hypertension 65.1%, 30%, and 43.9% can explained by the differences between the poor and non-poor individuals in the distribution of age, currently married, currently working, BMI, education, gender, living in a patriarchal family, no. of adult members, no. of household members, place of residence, and division respectively. ^3^ The difference between hypertension, as having undiagnosed hypertension, and untreated hypertension amongst poor-non-poor.

Gender and age both contributed to or helped widen the difference between the poor and non-poor in terms of the prevalence of undiagnosed hypertension. However, it was anticipated that BMI and current marital status would reduce the gap in the prevalence of undiagnosed hypertension. The respondents’ education helped to increase the prevalence of undiagnosed hypertension by 42.86% and had a positive effect on the difference between the poor and the non-poor.

## 4. Discussion

### 4.1 Major findings

This is the first study to demonstrate the poor-non-poor gap in the prevalence of hypertension, both undiagnosed and untreated, in Bangladesh. The following are the top seven conclusions: 1) a greater number of participants (25.1%) were found to have hypertension; 2) the prevalences of undiagnosed and untreated hypertension were 57.2% and 12.3%, respectively; 3) SES is important in predicting a person’s risk of developing hypertension and influencing a person’s risk of having undiagnosed and untreated hypertension; 4) age contributed to narrowing, whereas BMI was predicted to widen the poor-non-poor gap in hypertension prevalence; 5) when it comes to the risk of having undiagnosed hypertension, age, education, and gender have a role in the increasing gap between the poor and the non-poor; 6) BMI and current marital status contributed to narrowing the poor-non-poor gap in terms of the risk of undiagnosed hypertension; 7) Further, in terms of the likelihood of having untreated hypertension, age widened the gap between the poor and the non-poor **(Supplementary Figure 1)**.

### 4.2 Compare with other studies

Compared to a systematic review and meta-analysis carried out in Bangladesh (6), where the total pooled prevalence of hypertension was estimated to be 20.0%, the derived age-adjusted hypertension prevalence (25.1%) was higher. The age-standardized prevalence recorded in our study was lower than the 36.5% observed in South Asia for individuals aged 30 to 79 years (5). Additionally, the prevalence found in our research was also less than that reported for low- and middle-income countries (31.5%) and high-income countries (28.5%) among adults aged 20 years and older (29). Given that Bangladesh contributes significantly to the burden of hypertension in South-East Asia, the country’s startlingly high prevalence of hypertension is seen as a warning indicator of the disease’s quick expansion.

According to this data, the nation has low treatment-seeking behavior and a low diagnosis rate for hypertension. Additionally, the rate of undiagnosed and untreated hypertension in our study was like other previous studies conducted in resource-poor locations, such as Nepal (34.1%, 10.3%) (30), India (42.3%, 6%) (31), and Pakistan (37.7%, 25.7%) (32). These findings show that despite significant advancements, more treatment coverage is still required. Bangladesh needs to raise public awareness of hypertension considering these circumstances and provide appropriate education and follow-up for hypertension patients.

This study revealed intriguing relationships between hypertension prevalence and SES. Non-poor SES was associated with a higher prevalence of hypertension than poor SES, according to this study. We confirmed our findings with previous studies conducted in LMICs (18,19). However, in Western nations (14,15,16,17), the likelihood of having hypertension is higher in those with poor SES. When interpreting this contrast, it is important to consider the food security and energy expenditure patterns of individuals in poor SES in South Asia. These patterns include food scarcity, lower consumption of refined foods, and high energy expenditure due to moderate to intense physical activity at work (33). However, several studies, including meta-analyses, have revealed that poor socioeconomic groups in Western countries may be more likely than non-poor socioeconomic groups to smoke, have higher BMIs, and be less physically active, which could contribute to a higher prevalence of hypertension (34,35).

We found that patients with hypertension who belonged to the non-poor socioeconomic stratum had a higher likelihood of having their ailment diagnosed and treated compared to those in poor socioeconomic strata, which is consistent with previous studies carried out in LMICs (36,37). The higher diagnosis and treatment rates of hypertension among those with non-poor SES could be attributed to improved living conditions, easier access to hypertension information, and easier access to medication.

Based on the analysis of decomposition, age is one of the significant factors impacting the poor-non-poor gap in the prevalence of hypertension undiagnosed as having hypertension and untreated for hypertension. Negative coefficients (–0.0178 and –0.0052) indicate that age narrows the difference in the risk of hypertension and untreated hypertension between the non-poor and the poor. This implies that both poor and non-poor socioeconomic groups have an increased risk of hypertension and have untreated hypertension as they are older. However, age was found to positively affect the difference between the poor and the non-poor in terms of undiagnosed hypertension. It appears that in non-poor socioeconomic categories, aging is associated with a lower prevalence of undetected hypertension; this association was not seen in the poor SE groups. One plausible explanation is that elderly individuals from low-income households may not have known they had the sickness or may not have had the money to get the care they needed due to financial limitations (38). Therefore, policies aimed at providing older adults with low SES groups with better financial security under tailored subsidy schemes are one of the most crucial ways to close the age-related poor-non-poor gap in undiagnosed patients with hypertension.

Another important aspect of this study’s poor-non-poor gap in the prevalence of undiagnosed hypertension was the respondent’s level of education. A positive coefficient (0.0156) indicates that the prevalence of undiagnosed hypertension decreased with increasing education, favoring those with non-poor SES. Previous research has also demonstrated that this component is the main driver of the economic difference in the diagnosis of diabetes and utilization of eye care (39,40). Higher educated people are more likely to understand the advantages of having a hypertension diagnosis and to be able to control their blood pressure through early intervention and preventive care, according to research (41,42). Due to restricted educational opportunities and limited access to education, people in poor socioeconomic categories have greater difficulty in identifying and diagnosing hypertension. Therefore, to reduce socioeconomic differences in the diagnosis of hypertension, community-based education promotion programs can be helpful.

The results also demonstrate that, when it came to the likelihood of hypertension, a positive contribution of BMI (0.0579) implies that the prevalence of hypertension increased with BMI in non-poor socioeconomic categories. This conclusion can be supported by the fact that being overweight or obese raises the risk of hypertension (43) and that most overweight/obese individuals in Bangladesh come from non-poor socioeconomic backgrounds (44). However, it was found that a higher BMI was linked to a decreased likelihood of having undiagnosed hypertension in non-poor socioeconomic groups. This result is in line with other studies that demonstrate that participants’ awareness of hypertension is directly impacted by BMI (45).

This study also elucidated that gender contributed to widening the poor-non-poor gap (0.0093) in the prevalence of undiagnosed hypertension and that being male increased the likelihood of undiagnosed in the poor socioeconomic group. One plausible explanation for why men from low-income backgrounds are more likely to have undiagnosed hypertension could be that they face difficulties in managing their condition due to limited resources, which can limit their access to healthcare, education, lifestyle options, and healthcare knowledge. These results do suggest the possibility of gender-specific health interventions for early diagnosis to reduce the incidence of heart attacks and strokes due to high blood pressure, which may be preventable, particularly in the poor socioeconomic strata.

The results also showed that, for both poor and non-poor socioeconomic categories, marriage reduces the gap between the poor and the non-poor in terms of the prevalence of having undiagnosed hypertension. Marital status is the most important factor determining healthcare consumption, according to several previous investigations (46,47). Our results, which showed a decreased prevalence of undiagnosed hypertension among married people regardless of their SES, were in line with previous studies (48,49). Marriage may enhance the use of hypertension health services by altering lifestyle and health/illness awareness, as well as through facilitating diseases that are delayed or concealed because of societal stigma.

### 4.3 Strengths and limitations

The following are the study’s advantages: 1) since the results of our analysis are based on nationally representative data, they may be generalized to the population of Bangladesh; 2) clinical variables such as blood pressure, body weight, and height were measured using standardized techniques; and 3) PCA was employed to construct the household wealth index, yielding a more precise assessment of SES in Bangladesh than either income or consumption expenditures.

These are the study’s shortcomings: 1) since the participants were selected from the community, clinical record data, such as a history of hypertension and other diseases, was not investigated; 2) the study only gathered a limited quantity of information on the participants’ lifestyles. It covered characteristics linked to BMI but excluded smoking, other lifestyle factors, and food or exercise habits. Since making good lifestyle choices, such as eating a balanced diet, quitting smoking, and getting regular exercise, can reduce the risk of obesity and the development of hypertension, we have included overweight/obesity status in our analyses. Furthermore, considering the substantial and established links between hypertension and SES, it is unlikely that adding more lifestyle variables to the model will result in a non-significant relationship between predicting the likelihood of developing hypertension and SES; and 3) because this is a cross-sectional design, we were only able to collect each participant’s blood pressure data on a single day, making it unable to account for the ‘white coat effect’.

## 5. Conclusions

Bangladesh has a relatively high prevalence of undiagnosed and untreated hypertension, according to this study. The prevalence of hypertension was higher in non-poor SES groups than in poor SES groups, but individuals with hypertension in the latter group were also less likely to be aware of their condition and to obtain treatment. Our findings also suggested that BMI contributed to widening the poor-non-poor gap in terms of hypertension risk. Age, education, and gender contributed to widening the poor-non-poor gap in terms of the risk of undiagnosed hypertension. The findings of this study recommend that the government and other pertinent parties concentrate more on developing suitable policy measures for the early detection and ongoing treatment, particularly for older people, men, and those with lower levels of education who belong to poor socioeconomic groups. It will also be required to try to lower the prevalence of being overweight or obese among individuals in the non-poor SES category.

## Data Accessibility Statement

The data are available in a public, open access repository at https://dhsprogram.com/data/Access-Instructions.cfm.

## Additional File

The additional file for this article can be found as follows:

10.5334/gh.1372.s1 Supplementary Figure 1. Major findings.
