# Supplementary figures and images for: Decomposing the Poor-Non-Poor Gap in the Prevalence of Undiagnosed and Untreated Hypertension Among Bangladeshi Population
Source: Glob Heart. 2024 Dec 5;19(1):90. doi: 10.5334/gh.1372 (PMC11623077; doi:10.5334/gh.1372)

Supplementary figure 1: Major findings

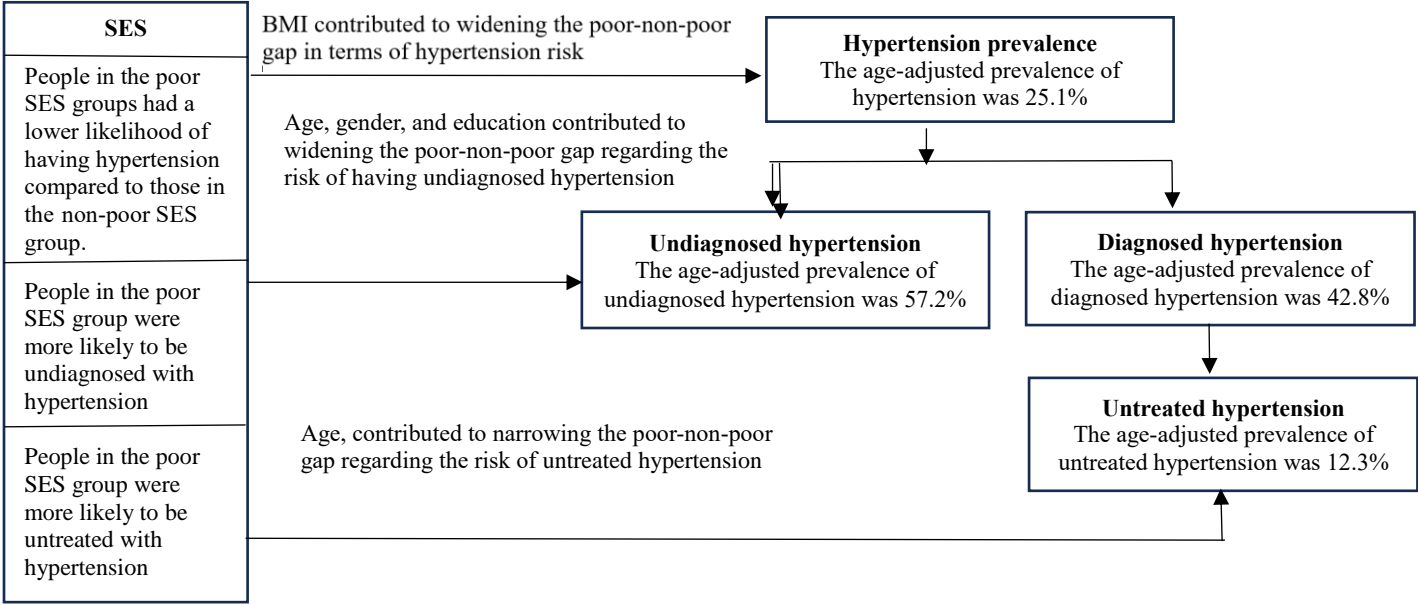

Supplement: Supplementary Figure 1. — Major findings. [file gh-19-1-1372-s1.pdf]
